# Cardioprotective Signaling: Outline and Future Directions

**DOI:** 10.3390/biomedicines13122973

**Published:** 2025-12-03

**Authors:** Aleksandar Jovanović

**Affiliations:** 1 Department of Basic and Clinical Sciences, University of Nicosia Medical School, Nicosia CY-1700, Cyprus; jovanovic.a@unic.ac.cy; 2 Center for Neuroscience and Integrative Brain Research (CENIBRE), University of Nicosia Medical School, Nicosia CY-1700, Cyprus

**Keywords:** cardioprotection, myocardial ischemia, ischemic preconditioning, reperfusion injury, signal transduction, protein kinases, hypoxia-inducible factor 1, microRNAs, connexins, ATP-sensitive potassium channels

## Abstract

Cardioprotection refers to the natural capacity of heart tissue to resist damage under conditions such as ischemia–reperfusion and various metabolic stresses. First identified in the phenomenon of ischemic preconditioning, the concept has since broadened to encompass other triggers of protective signaling, including hypoxia, temperature shifts, and a wide range of pharmacological compounds. This expansion indicates the presence of common molecular pathways and defense mechanisms. Known intracellular contributors to cardioprotection involve numerous factors, such as protein kinases, the reperfusion injury salvage kinase (RISK) cascade, the Survivor Activating Factor Enhancement (SAFE) pathway, hypoxia-inducible factor-1α (HIF1α), microRNAs, and Connexin 43, among others. These components are crucial in initiating downstream signaling, promoting the expression of protective genes, optimizing mitochondrial function, and regulating cytosolic and protein processes to maintain cardiac resilience. Key end-effectors include SUR2A, a regulatory subunit of sarcolemmal ATP-sensitive potassium (KATP) channels, autophagy, and mitochondria. Central mechanisms, such as modulation of the mitochondrial permeability transition pore and activation of KATP channels, play essential roles in the cardioprotective response. Although significant progress has been made in mapping these networks, many facets remain poorly understood. One of the most pressing challenges is to translate this knowledge into practical therapies and eventually create clinically applicable strategies to protect the heart.

## 1. Introduction

Cardiovascular diseases remain the leading cause of morbidity and mortality worldwide, with ischemic heart disease being a major contributor. Myocardial ischemia, defined as an imbalance between oxygen supply and myocardial demand, often results from the obstruction of coronary blood flow. The current therapeutic strategies primarily aim to restore myocardial perfusion (e.g., through thrombolysis, percutaneous coronary intervention, or coronary artery bypass grafting) and to decrease myocardial oxygen consumption by pharmacological means such as beta-blockers or nitrates. While these interventions have dramatically improved clinical outcomes, they are not sufficient to prevent ischemia–reperfusion (I/R) injury fully, and significant residual morbidity persists. This underscores the need for novel therapeutic approaches that can provide additional protection to the myocardium beyond reperfusion alone [1].

One such promising strategy is cardioprotection. Broadly defined, cardioprotection refers to the heart’s intrinsic and adaptive ability to withstand ischemia, reperfusion, and other metabolic or environmental stresses. The concept encompasses both endogenous protective mechanisms and externally induced interventions that reduce myocardial injury and improve functional recovery after ischemic events [2,3].

Over time, it became clear that a single pathway does not mediate cardioprotection, but rather by the convergence of multiple signaling networks involving ion channel modulation, reactive oxygen species (ROS) signaling, mitochondrial function, and anti-apoptotic cascades. Various humoral mediators, kinases, transcription factors, and mitochondrial effectors have been implicated in orchestrating this response [2,4]. Importantly, many of these mechanisms are conserved across species, suggesting that cardioprotection represents a fundamental biological adaptation aimed at preserving cardiac function under stress [4].

Today, cardioprotection is considered a highly attractive therapeutic concept. If successfully translated into clinical practice, it could complement reperfusion therapies, limit ischemic damage, preserve myocardial viability, and ultimately improve long-term outcomes in patients with ischemic heart disease. Nevertheless, despite promising preclinical data, clinical translation has proven challenging, with inconsistent results observed in human trials. Understanding the reasons for this gap and identifying robust cardioprotective strategies that are effective across diverse patient populations remain major goals of ongoing cardiovascular research.

## 2. Inducers of Cardioprotection

Cardioprotection refers to the ability of certain interventions to preserve the structure and function of the heart when it is exposed to ischemic or other types of injury. Over the past decades, a variety of procedures, environmental conditions, and pharmacological agents have been identified as inducers of cardioprotective responses. These strategies act through complex molecular pathways, ultimately aimed at reducing myocardial damage and improving cardiac recovery after ischemic events [1].

The first described cardioprotective strategy was ischemic preconditioning, a phenomenon discovered in the 1980s [3]. This procedure involves exposing the heart to brief, repetitive episodes of ischemia followed by reperfusion before a subsequent prolonged ischemic insult. Remarkably, this intervention was shown to significantly reduce infarct size and improve post-ischemic cardiac function. Following this discovery, researchers found that cardioprotection can also be achieved when brief cycles of ischemia–reperfusion are applied after a prolonged ischemic period. This process, termed ischemic postconditioning, has been demonstrated to attenuate reperfusion injury and preserve myocardial integrity [3].

Building on these observations, further studies revealed that cardioprotective effects can even be triggered at a distance from the heart. This phenomenon, known as remote ischemic conditioning (either preconditioning or postconditioning), is induced when transient ischemia–reperfusion cycles are applied to a remote organ or tissue, such as the limb. Signals generated at these distant sites are thought to be transmitted systemically, ultimately conferring protection to the myocardium [5].

Beyond ischemic interventions, environmental stressors have also been recognized as powerful modulators of cardioprotection. For instance, exposure to hypoxia or controlled fluctuations in oxygen tension can initiate adaptive mechanisms that enhance myocardial tolerance to subsequent ischemic episodes [6,7,8,9]. Similarly, temperature modulation, both in the form of hypothermia and hyperthermia, has been shown to exert cardioprotective effects. Hypothermia, in particular, is already applied clinically in specific contexts, such as after cardiac arrest, to limit ischemic damage [10,11].

In addition to physical interventions, a wide variety of endogenous and exogenous compounds have been identified as cardioprotective agents. Among these, mediators such as adenosine, nitric oxide, isosteviol, nicotinamide, and several growth factors have been demonstrated to reduce ischemic injury and promote cardiac recovery [12,13,14,15]. Many of these molecules act by activating intracellular signaling pathways that converge on key protective mechanisms, including reduced oxidative stress, inhibition of cell death pathways, and enhancement of mitochondrial function.

The diversity of methods and compounds capable of eliciting cardioprotection suggests that they either act through common signaling pathways or that multiple, parallel protective cascades can be recruited depending on the nature of the stimulus. This redundancy in cardioprotective mechanisms likely reflects the evolutionary importance of safeguarding the heart against ischemic damage, ensuring survival under adverse physiological conditions.

A summary of the major inducers of cardioprotection is presented in Table 1.

## 3. Intracellular Signaling of Cardioprotection

Over the last decades, numerous intracellular signaling molecules and pathways have been implicated in mediating cardioprotective effects. However, recent consensus statements and meta-analyses emphasize that the strength, reproducibility, and translational relevance of evidence for these pathways vary markedly. Despite considerable progress, there is still no definitive consensus on how these pathways are temporally coordinated, how they interact with one another, and which are indispensable for cardioprotection in humans. The complexity of intracellular signaling highlights the existence of redundant and overlapping mechanisms, which may serve as a biological safeguard to ensure myocardial survival under conditions of stress. Several major groups of signaling molecules have been repeatedly identified as key contributors, although their hierarchy and causal importance remain debated [25,35]

### 3.1. Protein Kinases

Protein kinases were among the earliest identified mediators of cardioprotective signaling. Protein kinase C (PKC), particularly the ε isoform, plays a central role in ischemic preconditioning and postconditioning, where it phosphorylates downstream targets to modulate mitochondrial function, ion channel activity, and apoptotic pathways. Importantly, not all PKC isoforms are protective, with some exerting neutral or even deleterious effects, underscoring the isoform-specific complexity of this signaling axis. PKC is often activated through pertussis-sensitive G protein-coupled receptors, which initiate cascades culminating in phosphorylation of cardioprotective proteins [34]. Nevertheless, meta-analytic evaluations reveal considerable variability in PKC-dependent cardioprotection across species and conditioning protocols, indicating that PKC signaling is highly context-dependent [37].

Other kinases, such as protein kinase A (PKA) and AMP-activated protein kinase (AMPK), have also been implicated. AMPK, in particular, acts as a metabolic sensor, maintaining energy balance under ischemic stress. Preconditioning has been shown to activate AMPK, which regulates the trafficking and opening of ATP-sensitive potassium (KATP) channels, thereby preserving mitochondrial integrity and reducing ischemic injury [31,38]. Hypoxia-induced cardioprotection, for instance, with exposure to 15% oxygen, also depends critically on AMPK activation [9]. However, the magnitude and necessity of AMPK activation appear to depend on the severity of ischemia, comorbidities, and energetic demand, raising uncertainty regarding its position in the overall hierarchy of cardioprotective kinases [39]. Additionally, p38 MAPK, extracellular signal-regulated kinases 1/2 (ERK1/2), and protein kinase G (PKG) are consistently linked with cardioprotective responses [40,41,42], although their contributions are not uniformly reproducible across models [43].

### 3.2. Reperfusion Injury Salvage Kinase (RISK) Pathway

A particularly well-characterized signaling entity is the RISK pathway, which integrates PI3K-Akt and MEK1-ERK1/2 cascades. This pathway is activated specifically at reperfusion, a time window when interventions can significantly alter infarct size. RISK signaling involves transactivation of the epidermal growth factor receptor and ultimately prevents opening of the mitochondrial permeability transition pore (mPTP), a critical determinant of cell death [44]. Although RISK has been widely reproduced in preclinical models, consensus reviews note that its robustness decreases in diseased or aged myocardium, indicating that upstream sensitivity is modulated by metabolic state [44]. However, controversy remains regarding the role of glycogen synthase kinase-3β (GSK3β), a downstream effector in this cascade. Some studies suggest its inhibition is essential for cardioprotection, while others report cardioprotection occurring independently of GSK3β regulation [19,44,45]. These conflicting findings underscore that GSK3β may act as a permissive rather than an obligatory mediator, with its relevance depending on species, comorbidities, or anesthetic background [35,44,46].

### 3.3. Survivor Activating Factor Enhancement (SAFE) Pathway

The SAFE pathway represents another cardioprotective signaling cascade, primarily activated by cytokines such as TNF-α at low concentrations. Central to this pathway is signal transducer and activator of transcription 3 (STAT3), which can function both in the cytosol and mitochondria. Cytosolic STAT3 acts as a transcription factor regulating cardioprotective gene expression, while mitochondrial STAT3 may directly influence electron transport chain function and ROS generation [25,47]. Despite accumulating evidence, the precise roles of STAT3’s subcellular localizations remain incompletely defined. Recent analyses suggest that SAFE does not function independently of RISK. Instead, significant cross-talk exists—STAT3 can modulate Akt activation, and RISK components can influence cytokine signaling—indicating that these pathways operate as parallel, partially redundant networks rather than isolated modules. However, their relative contributions vary across conditioning stimuli and disease states, complicating efforts to define a unified protective hierarchy [11,35].

### 3.4. Transcriptional Regulators and Noncoding RNAs

Hypoxia-inducible factor-1α (HIF1α) is a master regulator of adaptive responses to oxygen deprivation. By inducing genes that enhance glycolysis, angiogenesis, and antioxidant defenses, HIF1α not only shifts metabolism toward oxygen-efficient pathways but also improves mitochondrial performance and reduces oxidative stress. Recent evidence also suggests crosstalk between HIF1α signaling and noncoding RNAs, including microRNAs, thereby expanding the regulatory network of cardioprotection [22,24]. However, the reproducibility of specific HIF1α-dependent cardioprotective signatures across laboratories remains variable, and the translational viability of therapeutically targeting these pathways is still uncertain [23,48]. Indeed, multiple microRNAs (miRNAs) have been identified as modulators of cardioprotective signaling, regulating protein expression levels of kinases, mitochondrial proteins, and stress-response mediators [24]. However, the detailed mechanisms and therapeutic applicability of miRNA regulation in cardioprotection remain under active investigation, as many candidate miRNAs show cell-type–specific or context-dependent effects that complicate interpretation [32].

### 3.5. Mitochondrial Modulators

Mitochondria are central to ischemic injury and protection. Connexin 43 (Cx43), originally recognized for its role in gap junction communication, has been shown to translocate into mitochondria during ischemic preconditioning. Mitochondrial Cx43 influences KATP channel activity and modulates mitochondrial respiration and ROS signaling, making it a pivotal mediator of preconditioning responses [49]. Nonetheless, not all studies have reproduced the requirement for mitochondrial Cx43, and its abundance in human mitochondria remains debated in consensus documents [50,51]. Similarly, aldehyde dehydrogenase 2 (ALDH2), activated via PKCε signaling, confers resistance to oxidative stress by detoxifying reactive aldehydes generated during ischemia–reperfusion. Agents such as isoflurane and remote postconditioning have been demonstrated to activate ALDH2-dependent cardioprotection [52]. Yet, the magnitude of ALDH2-mediated protection appears to vary across genetic backgrounds, particularly in populations with ALDH2 polymorphisms, underscoring potential limitations for universal translation [53].

### 3.6. Stress-Responsive Proteins and Enzymes

Ischemic and hibernating myocardium upregulate various cytoprotective proteins, including inducible nitric oxide synthase (iNOS), superoxide dismutase (SOD), aldose reductase, and heme oxygenase-1 (HO-1). These proteins collectively function to modulate oxidative stress, improve cellular redox status, and maintain metabolic balance, thereby contributing to the cardioprotective phenotype [36]. However, these stress-responsive proteins are often downstream markers rather than primary initiators of protection, and their levels can vary substantially between models, limiting their utility as mechanistic endpoints [26].

### 3.7. Sirtuins and Metabolic Regulators

Sirtuins, a family of NAD^+^-dependent deacetylases, have recently emerged as key regulators of cardiometabolic stress resistance. Among them, sirtuin 1 (SIRT1) has been implicated in promoting autophagic flux, maintaining mitochondrial quality control, and enhancing biogenesis, all of which contribute to ischemic tolerance [54]. Despite strong mechanistic support, the reproducibility of sirtuin-mediated cardioprotection differs across experimental systems, and pharmacologic sirtuin activators have shown inconsistent effects in translational studies [55,56]. Other sirtuin family members are also under investigation for their roles in regulating oxidative metabolism and longevity-associated stress responses in cardiomyocytes.

**Figure 1:**
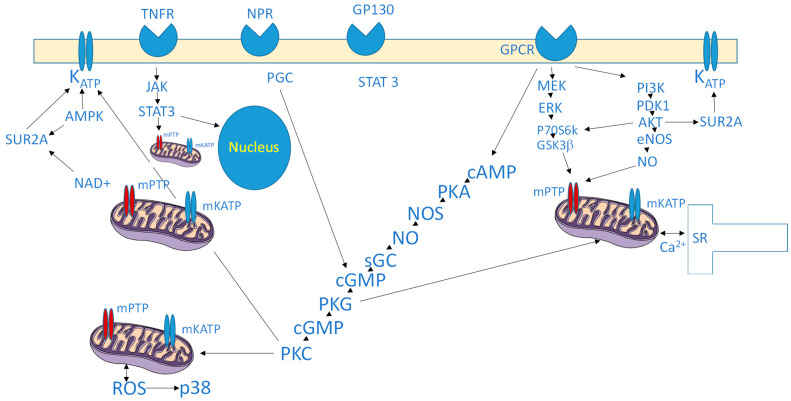
Representation of the major intracellular pathways involved in cardioprotection. It is not exhaustive, nor entirely precise, as several aspects remain debated or unknown. Nonetheless, the figure effectively conveys the intricate network of signaling events, although it does not reflect their precise timing or sequence. Abbreviations: Akt, protein kinase B; AMPK, 5′ AMP-activated protein kinase; BNP, cAMP, cyclic adenosine monophosphate; cGMP, cyclic guanosine monophosphate; ERK, extracellular signal-regulated kinase; GPCR, G protein–coupled receptor; gp130, glycoprotein 130; GSK3β, glycogen synthase kinase-3β; JAK, Janus Kinase, KATP, ATP-sensitive potassium channel; MEK, mitogen-activated protein kinase kinase; mKATP, mitochondrial ATP-sensitive potassium channel; mPTP, mitochondrial permeability transition pore; NAD, 3-phosphoinositide-dependent protein kinase-1; NO, nitric oxide; NOS, nitric oxide synthase; NPR, natriuretic peptide receptor; p70S6k, p70 ribosomal protein S6 kinase; PDK1, 3-phosphoinositide-dependent protein kinase-1; pGC, particulate guanylate cyclase; p38, p38 mitogen-activated protein kinase; PI3K, phosphatidylinositol (4,5)-bisphosphate 3-kinase; PKA, protein kinase A; PKC, protein kinase C; PKG, protein kinase G; ROS, reactive oxygen species; sGC, soluble guanylate cyclase; SR, sarcoplasmic reticulum; STAT, signal transducer and activator of transcription; SUR2A, sulfonylurea receptor 2A; TNFR, tumor necrosis factor receptor.

Taken together, these findings illustrate that cardioprotection is mediated by a highly complex and interconnected signaling network involving kinases, transcription factors, mitochondrial proteins, and metabolic regulators. While significant progress has been made in identifying these pathways, growing evidence emphasizes that RISK and SAFE pathways interact extensively and may compensate for one another, rather than functioning as isolated nodes. Further work is required to delineate the precise temporal sequence of their activation, the interplay between parallel cascades (Figure 1), their reproducibility across biological systems, and their relative importance in human disease settings.

A comprehensive overview of intracellular signaling factors implicated in cardioprotection is summarized in Table 2.

## 4. End-Effectors of Cardioprotection

While upstream signaling pathways coordinate the initiation of cardioprotective responses, their beneficial effects ultimately converge on a limited set of end-effectors that directly preserve cardiomyocyte viability during ischemia–reperfusion. These end-effectors function as the final executors of protection, translating intracellular signals into physiological outcomes such as stabilized mitochondrial function, reduced oxidative stress, preserved ionic balance, and maintenance of energy supply. Importantly, understanding the functional specialization of these end-effectors has revealed new therapeutic opportunities, including pharmacological activation, genetic modulation, and combinatorial targeting.

### 4.1. Mitochondria as Central End-Effectors

Mitochondria are universally recognized as the primary end-effectors of cardioprotection. Beyond their role as ATP generators, they are critical regulators of oxidative stress, apoptosis, and necrosis. Central to their protective role is the regulation of the mitochondrial permeability transition pore (mPTP), a nonspecific pore in the inner mitochondrial membrane that opens under conditions of calcium overload, oxidative stress, and ATP depletion. Persistent mPTP opening leads to mitochondrial swelling, collapse of membrane potential, and release of pro-apoptotic factors such as cytochrome c, culminating in cell death. Thus, inhibition or delayed opening of mPTP represents a cardinal mechanism of cardioprotection [29]. Another key mitochondrial target is the mitochondrial ATP-sensitive potassium (mitoKATP) channel, whose activation helps maintain mitochondrial homeostasis under stress. Opening of mitoKATP channels leads to mild mitochondrial depolarization, attenuation of calcium overload, modulation of ROS signaling, and preservation of ATP production, collectively reducing ischemia–reperfusion injury [30]. The functional importance of mKATP channels is distinct from that of sarcolemmal KATP channels: mKATP activation primarily stabilizes oxidative phosphorylation and moderates mitochondrial ROS bursts, whereas sarcolemmal KATP channels exert electrophysiological protective effects at the plasma membrane. Pharmacologic mitoKATP openers (e.g., diazoxide) have demonstrated potent preconditioning-like effects, although their clinical translation remains limited by off-target actions [60,61]. In parallel, mPTP inhibitors such as cyclosporine and emerging cyclophilin D-specific agents remain under investigation, despite mixed trial outcomes, as selective inhibition of mPTP opening remains a compelling therapeutic concept [35].

### 4.2. Sarcolemmal KATP Channels and SUR2A

In addition to mitochondria, sarcolemmal ATP-sensitive potassium (KATP) channels play crucial roles as end-effectors. These channels are formed by pore-forming Kir6.x subunits and regulatory sulfonylurea receptor subunits (SURs). SUR2A, the dominant regulatory subunit in cardiomyocytes, is dynamically regulated by cardioprotective signaling pathways. Enhanced expression of SUR2A increases cardiomyocyte resistance to ischemic injury, while its downregulation or inhibition blunts cardioprotection [62]. Both mitochondrial and sarcolemmal KATP channels, therefore, represent essential molecular determinants that couple upstream kinase signaling to electrophysiological and metabolic protection. Functionally, sarcolemmal KATP activation shortens action potential duration, reduces calcium influx, and decreases energy consumption. Strategies aimed at increasing SUR2A expression—such as gene therapy or pharmacologic SUR2A modulators—have gained interest as potential cardioprotective approaches [62].

### 4.3. Hexokinase 2 (HK2)

Hexokinase 2 (HK2), a glycolytic enzyme, also functions as an end-effector of cardioprotection through its interaction with mitochondria. HK2 binds to the outer mitochondrial membrane, where it stabilizes mitochondrial integrity, reduces ROS production, and prevents cytochrome c release. This localization is enhanced by preconditioning stimuli and is thought to be crucial in maintaining mitochondrial viability during ischemia–reperfusion stress [63]. HK2–mitochondrial binding also reduces mPTP sensitivity, providing a mechanistic link between metabolic regulation and mitochondrial stability. Modulation of HK2 binding—through upstream kinases such as Akt and ERK—has been proposed as a therapeutic strategy to reinforce mitochondrial resistance to ischemic stress [18].

### 4.4. Protein Nitrosation

Post-translational modifications such as S-nitrosation have also emerged as cardioprotective end-effectors. Nitrosation of mitochondrial and cytosolic proteins can modulate enzymatic activity, stabilize protein conformation, and limit oxidative damage. For example, nitrosation of complex I of the respiratory chain attenuates ROS bursts during reperfusion, thereby contributing to cardioprotection [27]. Nitrosation also interacts with signaling pathways controlling mPTP sensitivity and mitochondrial calcium uptake, revealing a complex functional network that may be pharmacologically targeted via NO donors or nitrosation-preserving agents [28].

### 4.5. Sarcoplasmic Reticulum and Calcium Handling

The sarcoplasmic reticulum (SR) is a central regulator of calcium cycling in cardiomyocytes, and perturbations in calcium homeostasis are a major contributor to ischemic injury. Cardioprotective interventions have been shown to modulate SR function through phosphorylation of regulatory proteins such as phospholamban, thereby enhancing calcium reuptake and stabilizing cytosolic calcium levels [16]. Preservation of SR integrity reduces calcium overload, prevents hypercontracture, and limits activation of calcium-dependent proteases during reperfusion. Agents that preserve SR–mitochondrial communication or enhance SERCA2a activity are being evaluated as therapeutic modulators of calcium homeostasis in the context of ischemia–reperfusion.

### 4.6. Cytoskeletal and Ionic Homeostasis

The cytoskeleton and associated mechanisms that regulate cell volume, ionic balance, and intracellular pH also act as important end-effectors. Stabilization of cytoskeletal elements maintains sarcolemmal integrity, while appropriate regulation of osmotic balance and ionic flux prevents cell swelling and rupture during ischemia–reperfusion. Maintenance of intracellular pH within tolerable limits is particularly important, as acidosis contributes to mPTP opening and protease activation [64].

### 4.7. Autophagy

Autophagy, the lysosome-mediated degradation and recycling of damaged organelles and proteins, plays a dual role in cardioprotection. During ischemia, autophagy is protective by removing damaged mitochondria and reducing the accumulation of toxic metabolic by-products. However, excessive or dysregulated autophagy during reperfusion can become detrimental, leading to loss of essential cellular components and autophagic cell death [33]. The timing and regulation of autophagy are therefore critical determinants of cardioprotective efficacy. SIRT1, an upstream regulator of autophagy and mitochondrial quality control, has emerged as a promising therapeutic target. SIRT1 activators (e.g., resveratrol and next-generation SRT compounds) enhance adaptive autophagy, promote mitophagy, and improve mitochondrial resistance to stress, offering a potential avenue for fine-tuning autophagy to maximize protection while avoiding excessive degradation [1].

In summary, cardioprotective signaling converges on a set of mitochondrial, cytosolic, and structural effectors that preserve cellular energy metabolism, ionic balance, and structural integrity during ischemic stress. By acting at the final execution stage, these effectors transform upstream signaling cascades into the tangible cellular outcomes of survival versus death. A deeper understanding of these end-effectors—along with targeted pharmacologic modulation of mPTP, KATP channels, HK2–mitochondrial binding, SR function, and autophagy—may provide the next generation of clinically actionable cardioprotective therapies.

The major end-effectors of cardioprotection are summarized in Table 3.

## 5. Clinical Implications and Patient Stratification

Translating cardioprotective strategies into clinical benefit requires recognition that patients vary substantially in their biological response to ischemia–reperfusion injury and to cardioprotective interventions. Several patient-specific modulators can profoundly alter the efficacy, magnitude, or even direction of cardioprotection.

Sex differences influence cardioprotective signaling at multiple levels. Pre-menopausal females often exhibit greater endogenous resilience to ischemic injury, partly attributable to estrogen-mediated modulation of mitochondrial function, nitric oxide signaling, and inflammatory pathways [67]. Conversely, males may benefit more from certain conditioning strategies, and sex-dependent responses have been observed for multiple pharmacological agents [68,69].

Age is another major determinant. Aging is associated with impaired mitochondrial dynamics, increased oxidative stress, diminished autophagic capacity, and a decline in pro-survival RISK and SAFE pathway signaling [17,70,71]. As a result, older patients often display attenuated responses to ischemic conditioning and pharmacologic cardioprotection. Preclinical models frequently underrepresent aged phenotypes, limiting translatability [17].

Metabolic status significantly modulates cardioprotection. Metabolic syndrome, obesity, insulin resistance, and especially diabetes blunt canonical cardioprotective signaling through chronic inflammation, endothelial dysfunction, altered mitochondrial substrate utilization, and impairment of protective ion channels such as mKATP [72,73]. Diabetic myocardium is also less responsive to both ischemic and pharmacologic conditioning interventions [73,74], emphasizing the importance of including metabolic comorbidities in mechanistic and translational studies.

**Figure 2:**
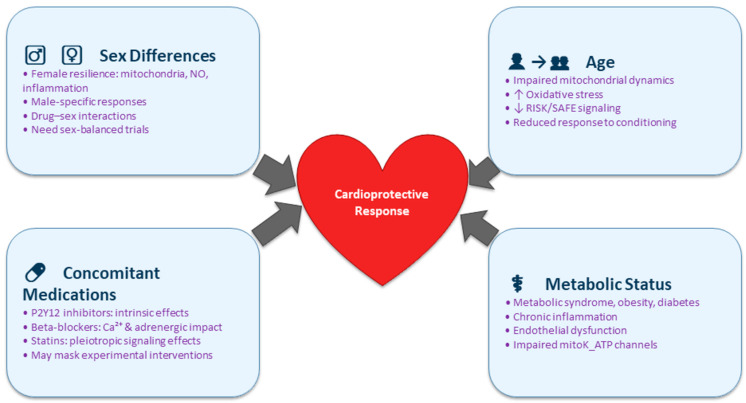
Translational barriers in cardioprotection. Cardioprotective efficacy is highly patient-dependent. Major modulators include sex, age, metabolic status, and medications. Biological heterogeneity affects response to ischemic and pharmacologic strategies. Precision cardioprotection requires stratified clinical trials. Tailored approaches may improve translation and therapeutic success.

Concomitant medications commonly prescribed to patients with acute coronary syndromes exert additional modulatory effects. P2Y_12_ inhibitors (e.g., ticagrelor) may possess intrinsic cardioprotective, anti-inflammatory, or mitochondrial-modulating actions [20,75,76]. Beta-blockers influence adrenergic signaling, mitochondrial calcium handling, and cytosolic kinase cascades integral to cardioprotection [77]. Statins have pleiotropic effects—including improved endothelial function, reduced oxidative stress, and modulation of cell survival pathways—that may interact synergistically or redundantly with experimental interventions [78]. Understanding these modulators is essential for patient stratification and precision cardioprotection. Future trial design should incorporate stratification by sex, age, and metabolic phenotype, with prespecified analyses accounting for background medical therapy. Tailoring cardioprotective interventions to patient-specific biological profiles may ultimately enhance therapeutic success and inform personalized treatment strategies in acute myocardial infarction and other ischemic conditions (Figure 2).

## 6. Future Directions

Despite remarkable advances in understanding the molecular and cellular mechanisms of cardioprotection, many critical gaps in knowledge remain. One of the most pressing challenges is to unravel the spatiotemporal dynamics of cardioprotective signaling networks. Although numerous kinases, transcription factors, and mitochondrial proteins have been implicated, their precise sequence of activation, subcellular compartmentalization, and interactions remains incompletely defined. Furthermore, cardioprotective responses are highly context-dependent, varying with the nature, duration, and severity of ischemia–reperfusion injury. Future studies should therefore employ advanced imaging, single-cell omics, and systems biology approaches to map cardioprotective signaling in space and time.

A major translational barrier lies in bridging the gap between experimental models and clinical reality. Most preclinical studies are conducted on young, healthy, genetically homogenous animals. In contrast, patients with acute myocardial infarction or other ischemic heart conditions often present with advanced age, metabolic syndrome, diabetes, hypertension, dyslipidemia, or prior cardiac injury, all of which may alter or blunt cardioprotective pathways. As already discussed, diabetes impairs AMPK signaling, reduces mitochondrial KATP channel function, and diminishes NO bioavailability; aging blunts RISK and SAFE pathway activation and reduces autophagic flux; chronic inflammation shifts kinase signaling away from pro-survival cascades; and endothelial dysfunction alters cGMP/PKG-dependent protection. Moreover, the concomitant use of medications such as antiplatelet agents, statins, or beta-blockers can modulate signaling cascades, sometimes masking or interfering with experimental cardioprotective effects. These translational barriers are reflected in several major clinical trials in which robust preclinical cardioprotective mechanisms failed to produce clinical benefit. For example, the CIRCUS trial evaluating cyclosporine for mPTP inhibition showed no reduction in infarct size or clinical outcomes, underscoring how delayed drug administration, comorbidity-related signaling impairment, and drug–drug interactions can negate mechanistic efficacy [65,66]. Likewise, the POSTEMI trial and the large CONDI-2/ERIC-PPCI trials found no benefit of remote ischemic conditioning, despite strong mechanistic rationale, likely due to heterogeneity in ischemia duration, anesthetic interference, background cardioprotective medications, and logistical challenges in precisely timing conditioning stimuli [21,79]. These examples highlight how cardioprotective signaling—although biologically robust—often becomes blunted, overridden, or mistimed in real-world clinical environments. The field must therefore prioritize the development of clinically relevant models that incorporate comorbidities, vascular dysfunction, inflammation, and polypharmacy to generate more reliable preclinical data. Additionally, future clinical trial designs must explicitly account for the sensitivity of cardioprotective pathways to timing and confounding pharmacological exposures, which have repeatedly undermined past trials [80].

Another underexplored area is the long-term impact of cardioprotective interventions. Most studies have focused on acute endpoints such as infarct size and short-term functional recovery. However, little is known about how cardioprotective signaling influences chronic outcomes, including myocardial remodeling, fibrosis, inflammation, arrhythmogenesis, and ultimately survival. Understanding how short-term protection translates into long-term benefits, whether it does so at all, will be essential for clinical translation.

The reproducibility and robustness of experimental data also remain concerns. Species differences can significantly affect cardioprotective responses, and some interventions that show efficacy in rodents fail in larger mammals or humans. Standardization of experimental protocols, multicenter preclinical trials, and greater transparency in reporting negative results will be necessary to identify truly reliable interventions.

Looking forward, several promising avenues emerge, but these must be better aligned with specific patient biology rather than framed generically:

- Mechanism-aligned personalized cardioprotection: Instead of broad personalization, future work should define which mechanistic defects dominate in specific patient groups. For example, diabetic patients may require AMPK-sensitizing or mKATP-targeting strategies; elderly patients may benefit from interventions that restore autophagy or augment SAFE signaling; patients on beta-blockers may need conditioning strategies independent of adrenergic pathways.
- Comorbidity-informed combination therapies: Combining pharmacological agents with ischemic conditioning or hypothermia should be tailored to patient-specific defects—e.g., pairing NO-donors with endothelial-dysfunction phenotypes; combining antioxidants with high-ROS aging phenotypes; or integrating SGLT2 inhibitors into conditioning paradigms for diabetics.
- Novel therapeutic targets matched to patient segments: Investigation into microRNAs, exosomes, mitochondrial dynamics regulators, or epigenetic modifiers should identify which targets compensate for pathway impairments unique to metabolic, inflammatory, or aging-associated states.
- Integration with tissue repair and regeneration: Because the capacity for repair varies across phenotypes (e.g., impaired neovascularization in diabetics, reduced progenitor activity in aging), the synergy between cardioprotective strategies and regenerative therapies should be evaluated in comorbidity-specific models.
- Mechanistically stratified clinical trial design: Future trials should incorporate prespecified strata based on mechanistic vulnerabilities—such as diabetes status, age-related mitochondrial dysfunction, or background medications known to activate overlapping pathways. Enrichment strategies (e.g., enrolling patients with clear evidence of residual RISK/SAFE pathway responsiveness), mechanistic biomarkers (e.g., NO-pathway activation, AMPK phosphorylation, mitochondrial function indices), and long-term endpoints (heart failure progression, arrhythmias, mortality) will be essential. Additionally, trials should control for drug–mechanism interactions by harmonizing background therapy or performing drug-stratified analyses.

Ultimately, the challenge for the field will be to transform mechanistic insights into clinically viable cardioprotective strategies that remain effective across heterogeneous, comorbid patient populations. Such strategies will likely need to be deployed as adjuncts to reperfusion therapy, enhancing its benefits while minimizing reperfusion injury. Achieving this goal will require close collaboration among basic scientists, translational researchers, and clinicians, as well as rigorously designed trials that reflect the complexity of real-world patients and the mechanistic diversity that determines their responsiveness [81].

## 7. Conclusions

In conclusion, although substantial progress has been achieved in elucidating the mechanisms underlying cardioprotection, a comprehensive understanding of this complex phenomenon remains incomplete. Research over the past decades has highlighted multiple signaling pathways, molecular mediators, and cellular processes that contribute to the heart’s ability to resist injury. However, the intricate interplay among these pathways, their context-dependent effects, and the influence of systemic factors such as age, comorbidities, and environmental stressors make it challenging to translate these findings into consistent clinical outcomes.

Moreover, despite promising preclinical studies, the development of effective and safe cardioprotective therapies for clinical use remains limited. Many potential interventions that show efficacy in experimental models have yet to demonstrate the same benefits in human trials, highlighting a critical gap between laboratory research and real-world application. This underscores the necessity for continued and focused investigation into the molecular and physiological mechanisms of cardioprotection, the identification of reliable biomarkers, and the design of innovative therapeutic strategies that can be effectively implemented in clinical practice. Advancing our understanding in these areas holds significant potential for improving patient outcomes, reducing the burden of cardiovascular diseases, and ultimately enhancing the quality of life for individuals at risk of cardiac injury.

## Figures and Tables

**Table 1:** Inducers of Cardioprotection.

Category	Inducer/Example	Mechanism(s) of Action (Proposed)
**Ischemic Procedures**	Ischemic preconditioning (brief ischemia–reperfusion before sustained ischemia)	Activates protective signaling cascades; reduces infarct size; improves post-ischemic recovery [3,5,16,17]
	Ischemic postconditioning (brief ischemia–reperfusion after sustained ischemia)	Limits reperfusion injury; reduces oxidative stress and apoptotic signaling [5,11,18]
	Remote ischemic preconditioning (transient ischemia at distant organ/tissue)	Release of humoral factors and neural signaling; systemic activation of cardioprotective pathways [19,20,21]
	Remote ischemic postconditioning	Similar to preconditioning; triggers systemic protective signals after prolonged ischemia [19,20,21]
**Environmental Stressors**	Hypoxia/intermittent hypoxia	Induction of hypoxia-inducible factors (HIFs); metabolic adaptation; angiogenesis [6,7,8,9,22,23]
	Altered oxygen tension	Modulation of mitochondrial function; activation of stress-response pathways [6,7,8,9,23,24]
	Hypothermia	Reduces metabolic demand; inhibits apoptosis and inflammation; preserves ATP [18,25]
	Hyperthermia	Heat shock protein (HSP) induction; cytoprotective protein expression [10,26]
**Pharmacological Agents**	Adenosine	Activates adenosine receptors (A1, A2); reduces calcium overload; improves coronary flow [2,12]
	Nitric oxide (NO)	Vasodilation; reduces platelet aggregation; modulates mitochondrial respiration [14,27,28]
	Isosteviol	Antioxidant and anti-apoptotic effects; modulation of mitochondrial KATP channels [15,29,30]
	Nicotinamide	Enhances NAD^+^ metabolism; supports mitochondrial function; reduces oxidative stress [31,32,33]
	Growth factors (e.g., insulin-like growth factor-1, vascular endothelial growth factor)	Promotes cell survival pathways; stimulates angiogenesis and repair mechanisms [4,13,34]
**Other Biological Mediators**	Heat shock proteins (HSPs)	Act as molecular chaperones; prevent protein misfolding; inhibit apoptosis [10,26]
	Cytokines and chemokines (e.g., TNF-α at low doses, interleukins)	Can activate pro-survival signaling cascades under controlled conditions [25,35,36]
	Endogenous opioids	Modulate receptor-mediated signaling; reduce excitotoxicity and calcium overload [4,25,35]

**Table 2:** Intracellular Signaling Factors Implicated in Cardioprotection.

Category	Molecule/Factor	Proposed Mechanism(s) of Action
**Kinases**	PKC (especially PKCε)	Isoform-specific activation; phosphorylation of mitochondrial/ion channel targets; modulation of apoptosis [5,11,34,37]
	PKA	Regulation of calcium handling and contractility; potential role in preconditioning [5,11]
	AMPK	Energy sensor; regulates trafficking/opening of KATP channels; supports metabolic adaptation to ischemia [9,38,39,43]
	p38 MAPK	Stress kinase; implicated in both protective and deleterious responses depending on context [4,11]
	ERK1/2	Part of RISK pathway; promotes cell survival during reperfusion [11,15,41,44]
	PKG	Downstream of NO–cGMP signaling; inhibits mPTP opening; vasodilatory effects [14,27,42]
	PI3K–Akt (RISK)	Enhances survival signaling; inhibits pro-death pathways; prevents mPTP opening [11,31,44]
	GSK3β	Downstream effector of RISK; role in cardioprotection remains debated [35,40,45]
**Transcription Factors & Cytokine Pathways**	STAT3 (SAFE pathway)	Activated by cytokines (e.g., TNF-α); regulates transcription of protective genes; modulates mitochondrial respiration [11,25,36,47]
	HIF-1α	Induces glycolytic shift, angiogenesis, antioxidant enzymes; interacts with noncoding RNAs [6,7,8,22,23,48]
**Noncoding RNAs**	MicroRNAs (miRNAs)	Post-transcriptional regulation of cardioprotective proteins; fine-tuning of kinase and mitochondrial signaling [4,13,24,32]
**Mitochondrial Modulators**	Connexin 43 (Cx43)	Phosphorylation and mitochondrial translocation; regulates KATP channels and mitochondrial respiration [19,49,50,51,57]
	Aldehyde dehydrogenase 2 (ALDH2)	Detoxifies reactive aldehydes; activated via PKCε; contributes to isoflurane and remote conditioning [5,52,53,58]
**Enzymatic Defenses/Stress Proteins**	iNOS (inducible nitric oxide synthase)	Generates protective NO signaling; modulates mitochondrial function [11,22,27,28]
	SOD (superoxide dismutase)	Detoxifies ROS; preserves redox balance [23,25,32,46]
	Aldose reductase	Maintains osmotic and redox homeostasis under ischemia [23,25]
	Heme oxygenase-1 (HO-1)	Generates antioxidant molecules (bilirubin, CO); cytoprotective role in ischemia [23,25,32,34]
**Metabolic Regulators**	Sirtuins (especially SIRT1)	NAD^+^-dependent deacetylases; promote autophagy, mitochondrial biogenesis, and metabolic resilience [33,54,55,56,59]

**Table 3:** End-Effectors of Cardioprotection.

Category	Effector/Component	Proposed Mechanism(s) of Action
**Mitochondria**	Mitochondrial permeability transition pore (mPTP)	Inhibition or delayed opening prevents mitochondrial swelling, cytochrome c release, and cell death [17,18,29,64,65,66]
	Mitochondrial KATP channels	Mild depolarization; reduced calcium overload; modulation of ROS signaling; preserved ATP production [29,30,57,60,61]
	Hexokinase 2 (HK2)	Binds mitochondria; stabilizes mitochondrial membranes; prevents cytochrome c release; reduces ROS [5,11,16,29]
**Ion Channels**	Sarcolemmal KATP channels (SUR2A subunit)	Modulate membrane potential and ionic flux; SUR2A expression enhances cardioprotection [6,7,8,9,31,38,62]
**Metabolic Enzymes**	ALDH2 (aldehyde dehydrogenase 2)	Detoxifies reactive aldehydes; reduces oxidative stress during ischemia–reperfusion [52,53,57,58]
**Post-translational Modifications**	Protein S-nitrosation	Modulates activity of mitochondrial/cytosolic proteins; attenuates ROS generation during reperfusion [11,22,27,38]
**Calcium Handling**	Sarcoplasmic reticulum (SR)	Modulation of calcium reuptake/release; phospholamban phosphorylation; prevention of calcium overload [16,23,24,57,58]
**Structural Elements**	Cytoskeleton, cell volume, ionic balance, pH stability	Preserve membrane integrity; prevent hypercontracture and cell swelling; stabilize intracellular pH [5,25,49,57,58]
**Autophagy**	Autophagy–lysosomal system	Removal of damaged organelles and toxic proteins (protective during ischemia); excessive activation may trigger autophagic cell death during reperfusion [11,23,32,33,57]

## Data Availability

No new data were created or analyzed. Data sharing does not apply to the article.

## Abbreviations

The following abbreviations are used in this manuscript:

ALDH2	Aldehyde Dehydrogenase 2
AMPK	AMP-Activated Protein Kinase
ATP	Adenosine Triphosphate
A1, A2	Adenosine Receptor Subtypes 1 and 2
cGMP	Cyclic Guanosine Monophosphate
CO	Carbon Monoxide
Cx43	Connexin 43
ERK1/2	Extracellular Signal-Regulated Kinases 1 and 2
GSK3β	Glycogen Synthase Kinase-3 Beta
HIF1α or HIF-1α	Hypoxia-Inducible Factor-1 Alpha
HK2	Hexokinase 2
HO-1	Heme Oxygenase-1
HSP/HSPs	Heat Shock Protein(s)
IPC/IPostC	Ischemic Preconditioning/Ischemic Postconditioning
I/R	Ischemia–Reperfusion
iNOS	Inducible Nitric Oxide Synthase
IPC	Ischemic Preconditioning
KATP	ATP-Sensitive Potassium (Channel)
MAPK	Mitogen-Activated Protein Kinase
MEK1	MAPK/ERK Kinase 1
miRNA(s)	MicroRNA(s)
mitoKATP	Mitochondrial ATP-Sensitive Potassium Channel
mPTP	Mitochondrial Permeability Transition Pore
miRNAs	MicroRNAs (noncoding regulatory RNAs)
NAD^+^	Nicotinamide Adenine Dinucleotide (oxidized form)
NO	Nitric Oxide
NO–cGMP	Nitric Oxide–Cyclic GMP Signaling Pathway
PI3K	Phosphoinositide 3-Kinase
PKA	Protein Kinase A
PKC	Protein Kinase C
PKCε	Protein Kinase C Epsilon Isoform
PKG	Protein Kinase G
RISK	Reperfusion Injury Salvage Kinase (pathway)
ROS	Reactive Oxygen Species
SAFE	Survivor Activating Factor Enhancement (pathway)
SIRT1	Sirtuin 1
SOD	Superoxide Dismutase
SR	Sarcoplasmic Reticulum
STAT3	Signal Transducer and Activator of Transcription 3
SUR2A	Sulfonylurea Receptor 2A (subunit of KATP channels)
TNF-α	Tumor Necrosis Factor Alpha
VEGF	Vascular Endothelial Growth Factor
